# Native Aortic Valve Endocarditis Associated With Methicillin-Sensitive Staphylococcus aureus Presenting With Spondylodiscitis: A Case Report

**DOI:** 10.7759/cureus.88650

**Published:** 2025-07-24

**Authors:** Lütfiye Kararmaz Aktaş, Emre Yılmaz, Sencer Çamcı, Şeyma Üngör

**Affiliations:** 1 Department of Cardiology, Giresun University, Giresun, TUR

**Keywords:** infective endocarditis, lower back pain, methicillin-sensitive staphylococcus aureus, nativ valve endocarditis, spondylodiscitis

## Abstract

Infective endocarditis (IE) is a public health problem that can present patients with complex clinical scenarios and carry a significant risk of mortality and morbidity. The ability of clinicians to analyze these complex clinical scenarios may be facilitated by the inclusion of relevant case reports and reviews in the literature. To this end, we sought to add this complex case report to the literature.

A 77-year-old man presented to the emergency department with a fever and widespread body pain, particularly in his chest, lower back, and buttocks. Initial investigations did not reveal any signs of IE. However, after ruling out other possible diagnoses, spondylodiscitis was detected via repeat lumbar magnetic resonance imaging. Methicillin-sensitive *Staphylococcus aureus* was identified in the control blood culture, and vegetation was identified in the control cardiac imaging.

Although laboratory and imaging findings cannot make a definitive diagnosis of IE in the early stages, repeated examinations can reach a definitive diagnosis in suspected cases. Therefore, clinicians should always consider endocarditis in patients with elevated acute-phase reactants and a clinical presentation suggestive of vascular or embolic phenomena.

## Introduction

Infective endocarditis (IE) is a rare but important public health problem with high morbidity and mortality. In 2019, the estimated incidence of IE was 13.8 per 100,000 people per year, resulting in 66,300 deaths worldwide [1]. The diagnosis of IE should be carefully considered in patients with predisposing risk factors associated with IE, heart murmurs, embolic phenomena, and vasculitis. Predisposing factors are divided into two categories: cardiac causes and comorbidities. Cardiac causes include a history of infective endocarditis, presence of cardiac implantable devices, ventricular assist devices, presence of prosthetic heart valves, bicuspid aortic valve, congenital heart disease, degenerative valve disease, bicuspid aortic valve, and hypertrophic cardiomyopathy [2]. Comorbidities include intravenous drug use, chronic liver disease, chronic kidney disease (especially dialysis patients), malignancy, corticosteroid use, advanced age, uncontrolled diabetes, immunosuppressed patients, and patients with indwelling venous catheters [3]. IE can have a variable clinical presentation. Therefore, its diagnosis is difficult and in the presence of risk factors, IE should be considered in all patients presenting with fever of unknown origin, sepsis, new murmur, vasculitis or embolic phenomena [4, 5].

Spondylodiscitis, which is one of the "hematogenous osteoarticular septic complications" included in the minor diagnostic criteria for IE, is the most common osteoarticular infectious complication in patients with IE, and its prevalence in patients with IE ranges from 2% to 10% [6]. In the diagnosis of spinal lesions, MRI is the preferred diagnostic modality for spondylodiscitis and vertebral osteomyelitis with 89-94% diagnostic accuracy [7]. MRI findings include edema of the vertebrae and discs, inflammation and/or abscess in the paravertebral/epidural area, increased gadolinium uptake in the vertebrae and discs, and bone deformation [7]. The most common symptom of spondylodiscitis is back pain. However, spondylodiscitis was found in 4% of patients with IE and back pain [7, 8]. We report a complex case of native aortic valve endocarditis presenting with spondylodiscitis.

## Case presentation

A 77-year-old male patient presented to the emergency department with fever and widespread body aches, particularly in the chest, lower back, and buttocks. The chest pain described by the patient at the initial presentation was noncardiac in nature; it was stabbing, worsened with movement, and spread throughout the chest. The patient's systolic and diastolic blood pressure was 125/72 mmHg, heart rate was 78/min, and temperature was 37.8°C. He had a history of coronary artery disease, hypertension, diabetes mellitus, and chronic kidney disease. Laboratory tests revealed troponin T: 0.587 µg/L (0-0.014), creatinine: 3.36 mg/dL (0.7-1.2), urea: 156 mg/dL (16.6-48.5), glomerular filtration rate: 16 ml/min/1.73 m2 (90-122), C-reactive protein: 2.36 mg/L (0-5), hemoglobin: 9.5 g/dL (13.5-17.5). The results from the hospital admission are presented in Table 1 under "admission"; the results from the nephropathy process are under "nephropathy"; the results from the endocarditis process are under "endocarditis."

**Table 1:** Hospital admission and laboratory results from different stages of the disease WBC - white blood cell; ALT - alanine aminotransferase; AST - aspartate aminotransferase; CRP - C-reactive protein

	Normal Range	Admission	Nephropathy	Endocarditis
Hemoglobin, g/dL	13.5 – 17.5	9.5	8.2	8.7
Hematocrit, %	41 – 53	29	24.3	25.8
Platelet, 10^9^/L	150 - 450	201	195	197
WBC, 10^9^/L	4 - 11	7.67	12.25	16.33
Glucose, mg/dL	75 – 99	231	283	263
Urea, mg/dL	16.6 – 48.5	156	200	161
Creatinine, mg/dL	0.7 – 1.2	3.36	5.48	4.72
ALT, u/L	0 - 41	8.91	10	12
AST, u/L	0 - 40	7.71	9	25
Sodium, mmol/L	136 - 145	139.78	133	133
Potassium, mmol/L	3.5 – 5.1	4.87	5.1	4.1
CRP, mg/L	<5	2.36	55.82	233.80
Troponin T, µg/L	0 – 0.014	0.587	0.745	1.92

Echocardiography revealed an ejection fraction (EF) of 45%, degenerative aortic valve, mild mitral and mild tricuspid regurgitation. The heart chambers (bilateral atria and ventricles), ascending aorta, septum, and posterior wall measurements were within normal limits. The inferior septum apical, anterior septum mid-apical, and apex were hypokinetic. On electrocardiography (ECG), the basic rhythm was sinus and the heart rate was 78/min. No specific ischemic findings were observed on the ECG. Due to chest pain and elevated troponin levels, the patient was admitted to the coronary intensive care unit with a presumptive diagnosis of non-ST-segment elevation myocardial infarction. Coronary angiography revealed an open stent previously implanted in the circumflex artery for acute coronary syndrome and non-critical stenoses in other coronary arteries (Video 1).

**Video 1:** Coronary angiography images

Medical follow-up was decided. The patient's pain did not improve after the procedure, and creatinine levels tended to increase. On the third day of hospitalization, the patient became anuric due to contrast nephropathy and was started on hemodialysis through a transfemoral dialysis catheter. The dialysis process was followed by the nephrology clinic during the hospitalization.

**Figure 1:**
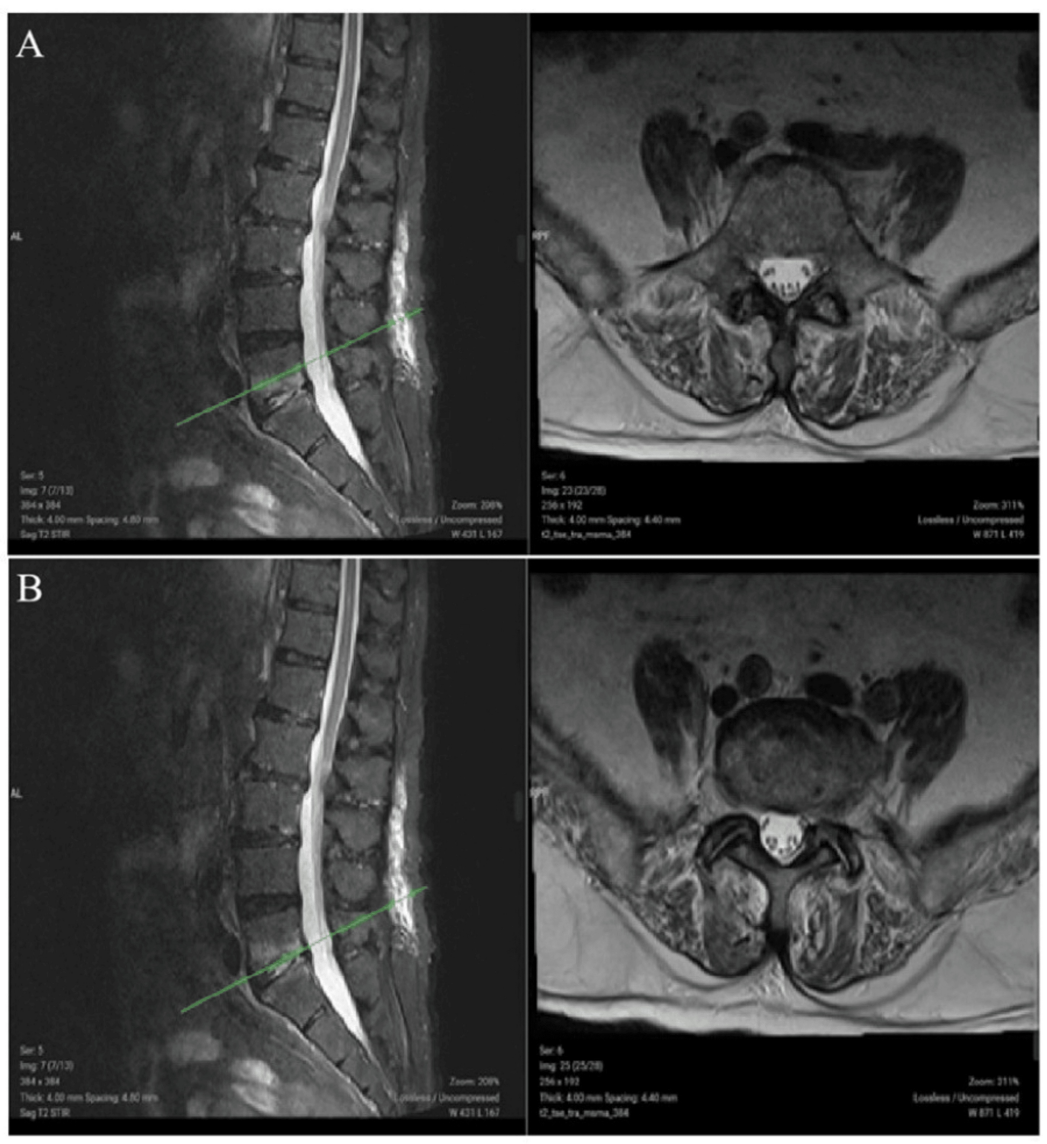
Gadolinium enhancement in the L5-S1 vertebral corpus (A) and intervertebral disc (B) observed on T2 short-tau inversion recovery sequence

During this time, his chest pain resolved, but his low back and hip pain worsened, his acute phase reactants increased, and he was referred to the departments of infectious diseases and physical medicine and rehabilitation. The patient was evaluated with a lumbar magnetic resonance imaging (MRI), and no findings were found to explain his symptoms. On the 9th day of hospitalization, the patient was started on teicoplanin, which was thought to be a catheter-related infection due to the continued increase in acute phase reactants. There was no growth in the blood culture. Echocardiography was repeated for infective endocarditis (IE), but no pathology was found to support IE. On the 12th day of hospitalization, the patient's low back and hip pain worsened; he developed difficulty walking and lumbar imaging was repeated. Comparison of the lumbar MRI with the initial MRI revealed inflammatory changes (gadolinium enhancement) involving the L5-S1 vertebral body and disc, consistent with spondylodiscitis (Figure 1). When the control blood culture grew methicillin-sensitive *Staphylococcus aureus* (MSSA), teicoplanin was discontinued and treatment with ampicillin-sulbactam was initiated.

**Figure 2:**
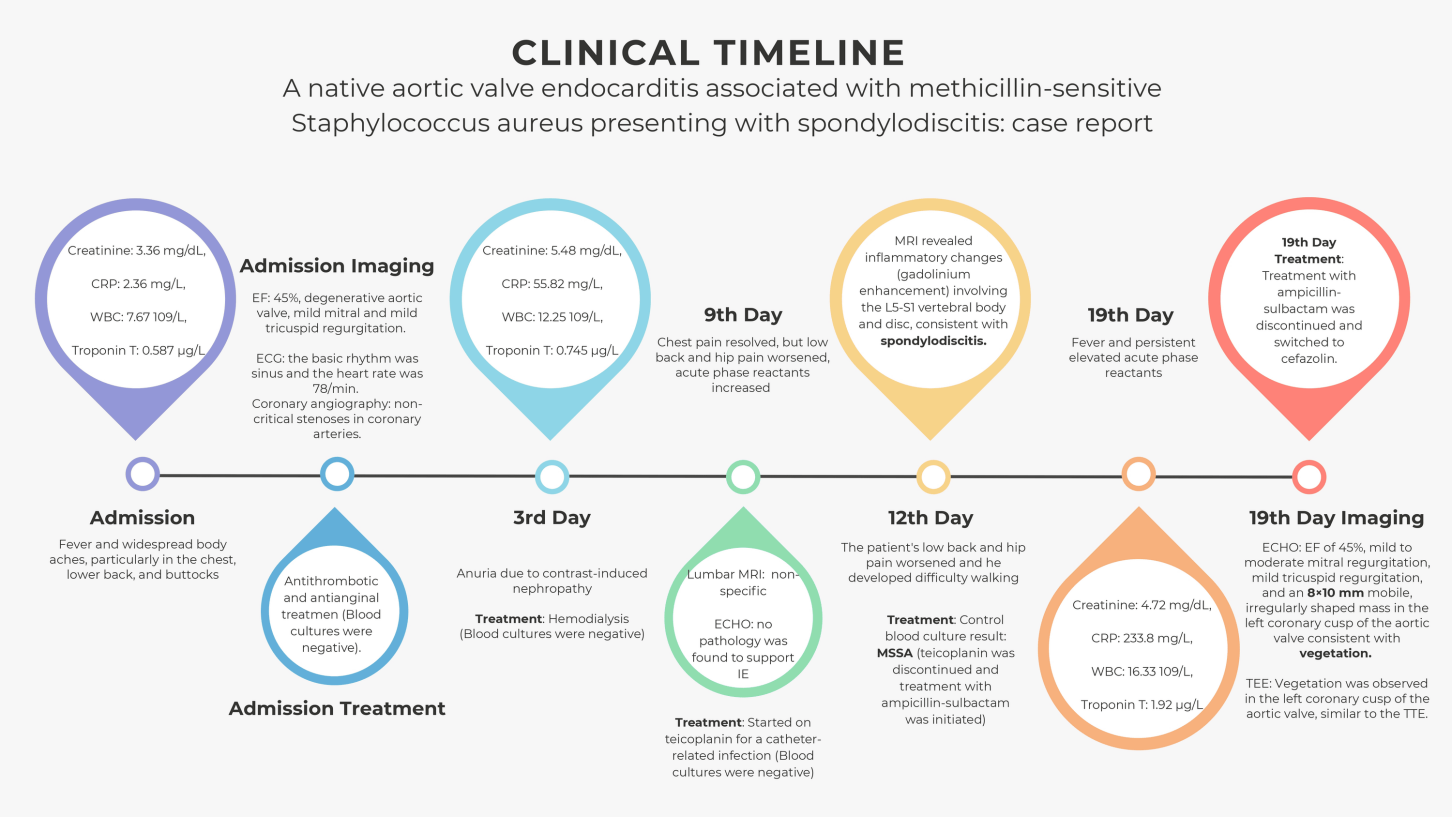
Clinical timeline CRP - C-reactive protein; WBC - white blood cell; EF - ejection fraction; ECG - electrocardiography; MRI - magnetic resonance imaging; ECHO - echocardiography; MSSA - methicillin-sensitive *Staphylococcus aureus*; TEE - transesophageal echocardiography; TTE - transthoracic echocardiography

On day 10 of antibiotic treatment and day 19 of hospitalization, echocardiography was repeated because of persistent elevated acute phase reactants. Echocardiography revealed an EF of 45%, mild to moderate mitral regurgitation, mild tricuspid regurgitation, and an 8×10 mm mobile, irregularly shaped mass in the left coronary cusp of the aortic valve consistent with vegetation. The patient was evaluated with transesophageal echocardiography, and vegetation was observed in the left coronary cusp of the aortic valve, similar to the transthoracic echocardiography (Video 2), and treatment with ampicillin-sulbactam was discontinued and switched to cefazolin. The clinical timeline of our case is shown in Figure 2.

**Video 2:** Video of transesophageal echocardiography showing a mass consistent with vegetation in the left coronary process of the native aortic valve

With continued antibiotherapy, inflammatory markers improved, blood culture showed no growth, and the patient was evaluated by a joint cardiology and cardiovascular surgery panel. Aortic valve dysfunction (insufficiency) developed and tended to progress, and aortic valve replacement surgery was planned due to failure of vegetation regression and persistence of spondylodiscitis symptoms. In the postoperative period, the patient had a normofunctional prosthetic aortic valve with EF 45% on control echocardiography and mild mitral and tricuspid regurgitation. The patient was discharged after six weeks of antibiotherapy, and conservative management was planned as there was no abscess formation associated with spondilodiscitis and the back pain resolved. At the three-month postoperative follow-up, it was observed that the symptoms of spondylodiscitis had completely resolved and there was no suspicious echocardiographic image or laboratory test in favor of endocarditis. 

## Discussion

There are some features that distinguish our case from similar cases. The association of native aortic valve endocarditis and spondylodiscitis is a rare clinical scenario. The number of publications reporting this association in the literature is limited [6,9]. Conversely, in our patient, it took time for the spondylodiscitis and endocarditis clinics to reach diagnostic consensus. Unlike other cases, we were able to obtain direct imaging findings only after the clinical scenario matured.

The etiology of IE varies depending on whether the valve is native or prosthetic. The most common organisms responsible for native valve IE are *Streptococcus viridians* and *Staphylococcus aureus*. Similarly, *Staphylococcus aureus* is the most common causative agent in the development of spondylodiscitis because this bacterium has highly invasive properties and can easily spread to the spine through the blood [2]. It has been suggested that spondylodiscitis is caused by microemboli of immune complexes [10]. Metastatic sites of infection typically occur in the lumbar vertebral region, where vascular density is highest, followed by the thoracic vertebral region [11]. The lumbar involvement in our case supports this.

MSSA is a pathogen that can cause serious and life-threatening systemic infections. It can have a variable clinical course and is associated with a high mortality rate if left untreated [12, 13]. The coexistence of two different infections, such as infective endocarditis and spondylodiscitis in the same patient, is rare. The fact that both infections are serious diseases that can spread by the hematogenous route and that their symptoms and signs may overshadow each other makes the management of these patients more complex and requires a multidisciplinary approach [9].

Successful treatment of IE is based primarily on microbial eradication with antimicrobial drugs. Surgical treatment is available to remove infected material and repair damaged valve structures. The goal of surgery for IE is to remove the infected structures and then restore anatomic and hemodynamic function. Repair or replacement of the affected heart valve(s) is performed depending on the degree of damage, severity of the disease, and patient characteristics. Aortic valve replacement is usually required for aortic IE. Aortic valve repair is very rare in the acute setting [2]. In our patient, aortic valve replacement was decided after a progressive increase in vegetation-related dysfunction of the aortic valve, which was initially not dysfunctional on control imaging.

We presented a case report of a patient with multiple comorbidities who presented with chest pain, fever, and diffuse body-lumbar-hip pain. None of the features considered risk factors for IE, such as history of previous IE, congenital heart disease, valve repair, prosthetic heart valve, implantable electronic cardiac devices, were present in our case. Nonspecific fever and the absence of cardiac risk factors for IE made the diagnosis difficult. In addition, because it took some time to obtain radiologic results to explain the symptoms and signs and to mature the clinical scenario, the diagnosis of IE and spondylodiscitis required patience, repeated imaging studies, and multidisciplinary dialogue. Elevated infectious parameters in laboratory tests and MSSA growth in blood cultures were the factors that helped us move toward the diagnosis and persist in our search. Positron emission tomography-computed tomography, which has recently been emphasized in the diagnosis of IE, could have been a time-saving imaging modality in our patient. The fact that we did not perform this imaging can be reported as a limitation. It has been shown in some case reports that this imaging not only provides diagnostic information, but also offers the opportunity to evaluate treatment efficacy [14, 15].

## Conclusions

Through this case, MSSA native aortic valve endocarditis and spondylodiscitis, we found that reaching diagnostic radiologic changes is possible with the maturation of the clinical scenario. We emphasize the importance of clinical follow-up, repeat imaging, and multidisciplinary decision-making in this process. In patients with multiple comorbidities, persistent elevation of acute phase reactants, presence of fever, even if nonspecific, and suspicious clinical presentation suggesting vascular/embolic phenomena, endocarditis should always be a diagnosis that clinicians should keep in mind.
